# Exposure Worry: The Psychological Impact of Perceived Ionizing Radiation Exposure in British Nuclear Test Veterans

**DOI:** 10.3390/ijerph182212188

**Published:** 2021-11-20

**Authors:** George Collett, William R. Young, Wendy Martin, Rhona M. Anderson

**Affiliations:** 1 Centre for Health Effects of Radiological and Chemical Agents, Institute of Health, Medicine and Environments, College of Health, Medicine and Life Sciences, Brunel University London, Uxbridge UB8 3PH, UK; wendy.martin@brunel.ac.uk (W.M.); Rhona.Anderson@brunel.ac.uk (R.M.A.); 2 Sport and Health Sciences, College of Life and Environmental Sciences, University of Exeter, Exeter EX1 2LU, UK; w.young@exeter.ac.uk

**Keywords:** radiation, anxiety, worry, British nuclear test veterans, GAI-SF, qualitative, exposure, mental health, guilt

## Abstract

Potential psychological issues faced by British nuclear test veterans have been under-researched. This study assessed the prevalence of clinically relevant anxiety in British nuclear test veterans and aimed to explore experiences of worry and the broader psychological impact of the British nuclear weapons testing programme. The Geriatric Anxiety Inventory (Short-Form) was completed by 89 British nuclear test veterans (33.7% met the criteria for clinically relevant anxiety). Nineteen veterans then participated in semi-structured interviews. Thematic analysis of the data generated three themes. The first theme highlighted how worry was relevant only in a few cases (four) generally regarding their grandchildren’s health, but the guilt in those who perceive responsibility for family health conditions also appeared to be a pertinent issue. The second theme highlighted the anger towards authorities resulting from perceived negligence and deception. The third theme highlighted the relevance of how certain life events across the life course influence the potential psychological impact. This study suggests that guilt must be considered in (potentially) exposed individuals whose family members experience health conditions, which may exacerbate distress. It also suggests the importance that authorities ensure transparency when dealing with any radiological exposure scenario to reduce the potential for anger.

## 1. Introduction

Following the Hiroshima and Nagasaki atomic bomb events which effectively ended the second world war, nuclear weapons testing proliferated. It is reported that some 22,000 men participated in the British nuclear testing programme between 1952 and 1958, including clean-up operations which extended into the 1960s in the Pacific islands and Australia [1,2]. Despite only 8% of the cohort receiving a non-zero dose (according to available dose records; [2]), there have been various media claims that exposure to ionising radiation has affected the health and quality of life in the veterans and in their descendants. While there is no convincing evidence of adverse physical health effects in this population [1,3,4,5], there remains the potential psychological impact of believing one has been exposed to ionizing radiation. Drawing on research involving other radiologically exposed populations, the objective of the present study is to highlight the potential for psychological effects in British nuclear test veterans, and to explore the potential psychological impact through a qualitative study.

The context of ionizing radiation exposure is inherently uncertain [6,7]. The ‘invisible’ nature of ionizing radiation including in the absence of any dosimetry, and the uncertainty regarding the potential future adverse health effects in the exposed individual and in their descendants, constitutes the psychological impact [6,7,8]. In this article, we regard the psychological impact as any thought process in relation to perceived ionizing radiation exposure. Worry (and the related affective experience of anxiety; [9]) appears particularly relevant to the uncertainty regarding possible exposure [6,8,10]. To elaborate, worry has been defined as a chain of negatively valenced thoughts regarding events that might happen in the future [9], and has been posited to be a cognitive problem-solving mechanism to prepare for uncertain future outcomes but containing negative outcomes [11,12].

Prominent work examining the psychological impact in exposed populations stemmed from Japanese atomic bomb studies, which highlighted the anxiety of acute radiation effects in themselves and of transmitting adverse health effects to subsequent generations [13,14,15]. Following the Three Mile Island accident, elevated biomarkers of psychological stress were observed in residents living near the power plant [16,17,18], demonstrating biological support for psychological effects. Importantly, the Chernobyl nuclear power plant accident and Fukushima nuclear power plant accident were marked by widespread psychological effects regardless of the dose received [6,19,20,21,22,23]. However, the psychological impact may not be limited to worry about ionizing radiation exposure, and it could be complicated by factors such as relocation/evacuation [24] and radical economic change [8], and stigmatization [25,26].

While there is a wealth of literature examining the psychological impact in contexts such as Japanese atomic bomb survivors and nuclear power plant accidents [8,13,14,23,27,28,29,30,31,32,33], few studies have been conducted with nuclear test veterans. For example, Murphy et al. [10] reported themes describing anxieties regarding the health effects of radiation exposure on United States (US) test veterans themselves and on their descendants, while an earlier study involving interviews with US test veterans reported a change in identity, worldview, and lifestyle by which veterans reported a lack of employment and loss of social relationships [34].

There is some recent quantified indication of anxiety issues in the British test veteran population. Firstly, Miles et al.’s [35] health needs audit from 2011 reported that 4% and 31% of their sample were “extremely anxious or depressed” and “moderately anxious or depressed”, respectively. While this indicates considerable mental health issues in British veterans of the testing programme, it is unclear the extent to which these issues are specific to depression, anxiety, or both. Secondly, Alexis-Martin et al. [36] reported roughly 8% of their sample of nuclear veterans self-reported themselves as having an anxiety condition, but an issue here is that there is the potential for clinically relevant (but undiagnosed) anxiety to go unreported. Finally, Dockerty et al. [37] found that 19% of their New Zealand nuclear veteran sample self-reported having anxiety. Collectively, these studies indicate that anxiety could be a pressing issue in the British nuclear test veteran population, but further examination using a validated anxiety measure and in-depth exploration is required.

Therefore, the aim of this study is to gain a quantitative indicator of clinically relevant anxiety in British nuclear test veterans, and to qualitatively explore worry about perceived ionizing radiation exposure and the broader psychological impact of being involved in the British nuclear weapons testing programme. A secondary aim is to explore how the potential psychological impact changes over the life course and whether certain psychological impacts are only experienced following specific life events.

## 2. Materials and Methods

### 2.1. Participants and Recruitment

Research packs were distributed to 246 test-veterans listed on a General Data Protection Regulation (GDPR)-compliant mailing list provided by the Nuclear Community Charity Fund (NCCF) in March 2019. A total of 146 of these were in the format of an online survey and 100 were postal. An advert with information about the study and contact details was also placed in the quarterly charity fund magazine. A total of 91 British nuclear test veterans (mean age = 81.68; aged between 74 and 90 years old) consented to participate in the study.

**Figure 1:**
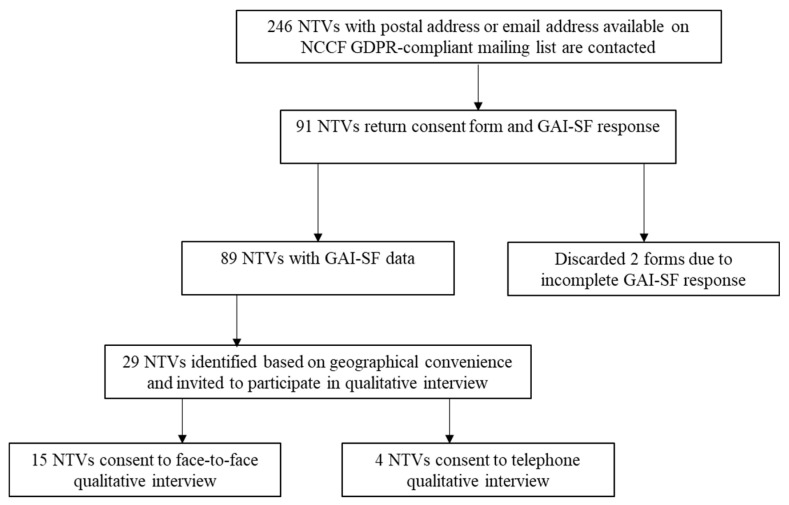
Flowchart for participant recruitment. NTVs = nuclear test veterans. NCCF = Nuclear Community Charity Fund. GDPR = General Data Protection Regulation. GAI-SF = Geriatric Anxiety Inventory–Short Form.

Of these, 29 nuclear veterans were subsequently invited to a face-to-face interview, based on geographical clusters for convenience. Fifteen agreed to participate in a face-to-face interview taking place in their home and a further four test veterans agreed to a telephone interview resulting in a total of 19 participants (Figure 1; aged between 75 and 89 years). Of these, 13 were stationed at Christmas Island (Kiritimati), four were stationed at Maralinga, one at the Montebello Islands, and one at Malden Island. Moreover, 14 of the participants witnessed one or more weapons tests.

### 2.2. Data Collection

Clinically relevant anxiety was measured using the Geriatric Anxiety Inventory – Short Form (GAI-SF): an anxiety screening measure validated for use in older adults [38]. The GAI-SF contains 5 agree/degree items which has been shown to have good convergent validity with more commonly used measures such as the State-Trait Anxiety Inventory-State subscale [38]. The 5 items comprising the GAI-SF are: “I worry a lot of the time”, “I often feel nervous”, “Little things bother me a lot”, “I think of myself as a worrier”, and “My own thoughts often make me anxious”. The total score for the GAI-SF ranges from 0 to 5. A cut-off score of 3 and above is an indicator of clinically relevant anxiety (e.g., generalized anxiety disorder; [38,39]).

Semi-structured interviews were conducted with 19 British nuclear test veterans to explore worry regarding perceived ionizing radiation exposure and the broader psychological impact of the British nuclear weapons testing programme. Seven of the nineteen interviewees met the criteria for clinically relevant anxiety (M = 2.11, SD = 2.00). The interviews ranged from 45 min to 2 h 17 min in duration and were conducted between August 2019 and January 2020. 

Each interview was biographical. To elaborate, each interview began by asking participants to describe their life in the few years leading up to their involvement in the testing programme, then to describe their experience during the programme, and to continue chronologically to present day with a focus on life events relevant to mental health or any psychological impact (e.g., thought processes or related emotions). The interviews also loosely followed a set of pre-determined open-ended questions designed to cover six topics chosen as areas of interest: identity, uncertainty (worry), risk perception, health, subsequent life events, and cognitive function. Each of these six topics were probed at relevant points during the interviews. For example, when participants were describing their experiences during the testing programme, risk perception would be probed by asking “Do you think there were any risks to taking part in the programme?” and followed by “How do these risks make you feel?” to explore any psychological impact related to perceived risk. Naturally, not all the topics arose during the participant’s biographies. In which case, the topics were probed at the end of their biographies to elicit further interview data.

Since the interviews relied heavily on the recall of past events, face-to-face participants were encouraged to have ready photographs significant to their testing experience; a method influenced by photo-elicitation [40]. Some participants presented photographs taken during the testing programme and from medical imaging of descendants depicting certain health conditions, while other participants presented documents such as health reports, newspaper clippings, brochure-type documents from their service, and safety protocols. While participants can find meaning in objects or photographs which is thought to facilitate discussion regarding potentially sensitive topics [41], the use of objects or photographs may help elicit emotionally laden memories of significant events occurring many years ago [42].

### 2.3. Analysis

A reflexive diary was kept by the lead researcher which included thoughts pertaining to potential themes and any key interactions between participants and any family members present during the interview. The interview recordings were transcribed verbatim and analysed using thematic analysis [43]. Specifically, interview transcripts were coded by hand using highlighter pens and writing the code labels in the margin of the transcript. Sections of the transcript were coded with limited interpretation of the text; thus, an inductive approach was taken to the analysis. The codes were generated for varying lengths of transcript segments, ranging from single sentences to a full paragraph depending on the content. For example, a paragraph presenting five distinct aspects would generate five codes, but of course interview responses may not be perfectly structured or concise, therefore a paragraph may also only generate one or two codes. After all initial codes were completed, patterns (themes) across the coded transcripts were searched by using the coloured highlighters as a visual indicator. Themes and their illustrative quotes were then compiled into tables in Word Software. 

## 3. Results

**Figure 2:**
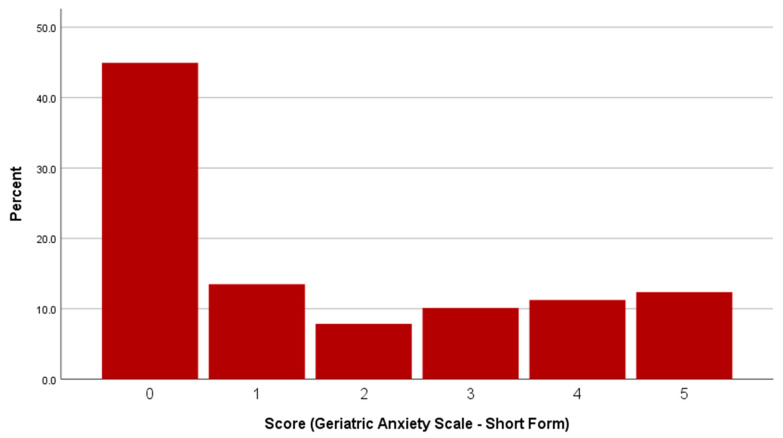
Distribution of nuclear test veteran scores on the Geriatric Anxiety Scale (Short-Form) (*n* = 89).

Out of the 89 valid responses, 30 (33.7%) participants met the criteria for clinically relevant anxiety. A mean GAI-SF score of 1.66 (SD = 1.88) was obtained. Figure 2 shows the distribution of scores for the 89 responses. 

The thematic analysis of the 19 interview transcripts generated three interconnected themes giving a rich description of the verbal data in relation to the psychological impact, namely “worry, responsibility, and guilt”, “us vs. them”, and “change across the life course”. At the time of the interview, the participants generally reported not being worried or anxious about their exposure in the context of future adverse health effects in themselves, but there were some instances of worry regarding their family members’ health. While worry was the central topic to be explored, it was apparent that other psychological effects marked by guilt and frustration were described by some participants, and that these were not limited to the perceived possibility of adverse health effects. Each theme will be explored in detail to provide an in-depth understanding regarding the complicated nature of the potential psychological impact of being involved in the British nuclear testing programme across the life course.

### 3.1. Worry, Responsibility, and Guilt

This central theme captures participant discussions regarding any perceived psychological impact (or lack of) of the British nuclear testing programme. Indeed, lay people are likely to report emotions when describing their thought processes, and participants were sometimes asked “how does it make you feel?” as a convenient way of engaging participants in describing the psychological impact. Therefore, reports of feelings or emotions were acknowledged in the generation of themes. The present theme consists of two subthemes: “worry” and “responsibility and guilt”.

#### 3.1.1. Worry 

Generally, the veterans reported not being worried about their own health. One reason for the limited worry about their own health is their chronological age. For example, four veterans alluded to how they had reached an age where they should not be worrying about their own health. 

Interestingly, there was also limited worry about their children’s future health. The reason appeared to be linked to the life course: the fact that, generally, children of the participants had developed without serious health conditions and any children with serious health conditions had been managed.

Rather, any worry tended to be directed to the participants’ grandchildren, which was evident in four veterans. To illustrate, Veteran M, a veteran involved in the clean-up operation at Christmas Island, describes how he was “past that stage” regarding worrying about his own health following radiation exposure, but the focus of the worry was related to the potential for “carrying on” health effects to his grandchildren:

“I’m 81 and the other ones didn’t even saw that age, you know? We’re getting back to the beginning. It’s when you read about the one that kidneys or something, the kidneys were welded together when it was born and things like that, and the deformity in kids and things like that. I wouldn’t like the think that was my grandchildren, and I wouldn’t like to think- That’s what worries me. The carrying it on.” (Veteran M).

The severity of the worry about the health of grandchildren varied across the participants, and in some cases, this was limited and phrases such as “on the back burner” exemplified this. 

Of course, not all participants had grandchildren. Naturally, there was no present concern for the health of grandchildren in the few veterans who do not have grandchildren, but two veterans acknowledged that the potential for their descendants to have children would otherwise bring concern to the forefront. For example, Veteran F, who served at Maralinga, stated that although he would be reluctant to say it, he was pleased that he did not have grandchildren due to the potential for passing on health effects. 

#### 3.1.2. Responsibility and Guilt

The second subtheme pertains to the notion of responsibility and the related feelings of guilt. The term “responsibility” was not explicitly mentioned in the data but was implicitly referred to by participants’ suggestions that their involvement in the tests were a contributing factor to any serious health effect in family members. 

In cases where descendants had a serious physical health condition, there appeared considerable psychological effects in the participants who perceived themselves as being responsible. An extract from a telephone interview with Veteran Q illustrates such effects:

“I do wonder about certain things in life. Um I mean my daughter had breast cancer, then she’s got tumours on the brain and then she died. Whether that was anything to do with it because I’ve read so many times that it’s not always the people who witnessed the test, it be the generation after that are affected. Also had a granddaughter with Katz disease, um that was a rare disease of the nervous system so you worry about these things whether it’s a contributing factor or not, but there’s nothing you can do about it obviously but you just can’t help wondering about it.” (Veteran Q).

Later in the interview, Veteran Q stated that “until the day I die” he will “always wonder” about whether his involvement in the testing programme was responsible for their deaths. Indeed, thoughts about whether one is responsible for another’s death occurring in the past can be regarded as a worry, indicating that notions of responsibility for past events and worry could be linked.

This notion of responsibility was further evident in participants who had descendants surviving with serious health conditions, but also applied to their wives with health conditions. Of note, the language used explicitly refers to specific emotions related to perceived responsibility, primarily the experience of guilt. For example, Veteran F described the perceived responsibility and guilt regarding his wife’s mental health resulting from ectopic pregnancies:

“Deep down I’m very angry. Anger and guilt rather than frustration. Guilt. Because I know you say it isn’t my fault and yes I understand it isn’t my fault but I can’t convince myself it isn’t my fault. Can you understand that? It sounds totally illogical, but I can’t.” (Veteran F).

Continuing along this line, three veterans would ask themselves how life may have been different if they were not involved in the testing programme:

“No I mean, when my wife died we would’ve had another two years we’d have had our diamond wedding and it just goes through my mind “only if I hadn’t gone to Montebello would we have seen our 60 years?” (Veteran K).

To summarise the current psychological impact of ionizing radiation exposure in these veterans, it appears that, generally, there is limited worry about the potential future adverse health effects on themselves. Indeed, most of the participants were not particularly worried about the future adverse health effects in their family members since most of those with a health condition had been effectively managed, but there were some instances where worry persisted (particularly regarding grandchildren). Beyond this, there is the perceived responsibility for their family’s health conditions which brings feelings of guilt to participants who perceived them to be affected.

### 3.2. Us vs. Them

The second theme describes the antagonism between the British nuclear test veteran community and authoritative groups, primarily the government and scientists involved at the time. While this theme pertains to a psychological impact, this theme was generated particularly in relation to socio-psychological impact of authoritative groups. This theme is comprised of two subthemes. The first subtheme, “power dynamic”, describes the power dynamic relating to perceptions of experimentation, as part of scientists’ and the government’s effort to understand the impact of nuclear weapons on buildings and on humans (i.e., the veterans). The second subtheme, “recognition”, goes further and describes the challenge for recognition from the government. Ultimately, the perceived role of authorities led to strong feelings of anger and frustration in five of the participants, contributing to the broader psychological impact of the testing programme. The following extract from Veteran Q is positioned here because it effectively captures the perceptions of an “us vs. them” notion in the test-veteran population:

“It brings back things like ‘us and them’. You hear about it and you see about it so, so commonly, um really on something like that which can have such disastrous effect on people you’d have thought everybody would be treated as a standard, by the same. But I didn’t know that at the time. It’s only something I’ve read in the last couple of years with these various articles I get but um you get bits of information keep coming out but it’s just uh bearing in mind what we had to go through or what could’ve happened that we’d all be treated the same.” (Veteran Q).

Importantly, the above extract describes the disappointment due to perceiving themselves as being marginalised by authorities with power, namely the government. This perception leads to the first subtheme: 

#### 3.2.1. Power Dynamic

There were two general perceptions on the role of the government or, as labelled by two veterans, “the powers that be”. This perception differed primarily between intention and negligence. Many veterans perceived themselves to be victims of experimentation or concealed information by the authorities regarding the risks of nuclear weapons. For example, the label “guinea pig” was used by seven veterans and seemed to constitute an identity:

“My view of a nuclear test veteran is that we’re all members of the mushroom club, kept in the dark, shovelled shit from time to time. And that plaque up there tells you what else we are. Guinea pigs. Guinea pigs. That’s what we are. That’s my view of what nuclear test veterans are. A bunch of people who haven’t a clue what they are doing who were sent out there as guinea pigs to work on tests. They would never have sent us to the forward area where there’s nothing to pick up, to pick up bits, if that hadn’t been part of being guinea pigs and being part of the mushroom club.” (Veteran F).

Drawing on this, there were also instances where aspects of the testing programme were interpreted suspiciously. For example, Veteran O mentioned the large number of veterans required on the island for a (perceived) complex but superfluous infrastructure. Following this description, he suggested that it could be interpreted under the notion of “guinea pigs”. Moreover, in one case, regular medical check-ups following their service, which could be perceived as routine, were perceived as monitoring health under the intention of examining effects of radiation exposure on the person. 

There were also perceptions regarding limited information and communication of health risk at the nuclear testing programme. Phrases such as “kept in the dark” were also used to describe the lack of radiation dosimetry available on Christmas Island. Veteran O, who referred to a large Atomic Weapons Research Establishment booklet of what the protocol should have been for radiation protection, he suggested that the combination of not being told anything about risk and the possibility of punishment for disobeying orders during national service were two factors which led to veterans entering high risk areas without appropriate protective clothing. Veteran S commented on the fact that he was also given very limited information in his previous postings with the RAF in Libya and Egypt, indicating that limited information is a norm in the armed forces. 

Relating to the perceived power dynamic, four veterans highlighted a perceived lack of choice, which appeared to be partly related to national service. Indeed, the characteristics of doing as you are told in the armed forces, and perceptions of being withheld information, and ultimately the limited control, can lead to feelings of being victimised. The notion of being a “victim”, that is, the risks of radiation being imposed on veterans with little choice, may also form a significant component of the test-veteran identity:

“And if you say how do I identify with them, I identify as one of the 160 victims. Because we weren’t given a choice.” (Veteran A).

#### 3.2.2. Recognition

Forming the second subtheme is the notion of recognition. Seven veterans expressed disappointment towards the government and the perceived lack of recognition and, in some cases, they felt “forgotten” or “erased”. In fact, the issue of recognition was the most widely reported topic across the interviews and even featured in participants who did not perceive themselves to be adversely affected by ionizing radiation exposure. Nonetheless, issues regarding recognition, compensation, truth, and gratitude were evidently salient across the interviews. The need for recognition was always discussed in relation to the government, but what specifically was to be recognised varied between the veterans. For some, recognition meant acknowledgement for their service, while for others it meant admitting negligence and, in some cases, deception. 

One way in which recognition might be gained is through the provision of a medal. It appeared that the tangible aspect of a medal was not significant to most of the veterans, but what was important was what the medal symbolised, which is gratitude for participating in the testing programme. Furthermore, it was important to veterans that the UK government acknowledges and accepts that the nuclear testing programme occurred, and for some, that veterans were adversely affected by ionizing radiation exposure:

“All I want. All I want-I’m not too worried about a medal. I mean it would be nice to have a medal because we’ve served our country probably as much as some of the people in the minor infringements that went on. But having said that, all I want is the British government to say, “yes we accept that” and the families around, perhaps give them a widow’s pension so that they look after the families that are suffering because of it. If we can prove that.” (Veteran F).

The above extract is also one example of how two veterans showed awareness that other individuals had been awarded medals for non-combat expeditions and question why they had not received the same appreciation. In addition, most of the veterans also compared the UK to other governments of countries such as France, Australia, and the USA, who had compensated their veterans for their respective nuclear weapons testing programmes, thus implying recognition for their service. Generally, financial compensation was not of great importance, but they did state that they would like compensation to be awarded to those veterans and their families who have been affected by ionizing radiation exposure.

The issue of recognition appeared inextricably linked to compensation. When the veterans were asked why the government had not recognised or admitted that some veterans were adversely affected by the testing programme, the consensus was that recognition is inextricably linked to compensation, which would be a financial burden for the government. Moreover, the veterans believed that compensation would therefore symbolise that the government have inflicted harm on the veterans through negligence (e.g., perceived inadequate protective clothing). Three veterans would comment on the fact that they are an aged cohort and as a result there are not many surviving, alluding to the idiom “running down the clock”. Specifically, it was sometimes perceived that the government was waiting for the veterans to die so that a reduced pay-out for compensation might be given; or that the issue of admission became less relevant to the government, if there are no test veterans alive. 

Gaining admission was noted as particularly challenging given that the present-day government and scientists had no role in the nuclear weapons testing programme. The following extract illustrates the negative attitudes towards authorities, drawing on notions of truth and morality:

“No one has had the decency to say “yes, we did fail in that respect.” No one said that. And that really is the cause that I feel is really, really bad for a modern nation like this and our government still looked back on the old paperwork and says oh yeah, yeah, yeah. But they’re reading lies. They’re reading lies that the scientists have told them. Scientists know what’s going off. They’re the ones that pulled all the strings in Australia. Everything that happened in Australia, don’t know about Christmas Island, I wasn’t involved with that. But Maralinga was solely controlled by those people. And the Australian government was controlled to them. If they could lie to them then they certainly could lie to us. To this day no one has owned up.” (Veteran I)

### 3.3. Change across the Life Course

Any worry and the broader psychological impact (or a lack of psychological impact) relating to possible adverse health effects, and the socio-psychological impact relating to antagonisms between the veterans and the government, was not necessarily consistent throughout life. This theme describes the role of time in the psychological impact, with reference to certain personal and societal events, and is categorised as two subthemes. The first subtheme, “the tests”, describes the historical and social context of when the nuclear testing programme took place as an explanation for the limited extent of any worry over time. The second subtheme, “after the tests”, describes the timing of subsequent events and their relevance in understanding the possible psychological impact after the testing programme.

#### 3.3.1. The Tests 

Most of the participants had generally positive experiences at the time of the testing programme. The veterans suggested their age at the time as an explanation of why they had no initial worry for any potential consequence, and often described a perceived limited understanding of the world:

“Like I said, one of the chaps must’ve said “oh yeah, yeah they do H bomb testing” which didn’t mean a lot really to an 18-year-old. It was just that we were going to a lovely, little island in the south pacific. Nice weather, and all that, you know.” (Veteran N).

The prospect of visiting a foreign land with hot weather was described by some veterans as an easy life, with leisure activities at the weekends. The terms “naïve” and “ignorant” were also used by four veterans to describe their perceptions at the time of their service. Two veterans used the proverb “ignorance is bliss” when detailing the extent of their worry. Similarly, three veterans said that, while they were aware of what a nuclear bomb meant because of their knowledge of the atomic bombings of Hiroshima and Nagasaki, this had limited significance to them in terms of risk. Some veterans would describe the anticipation and excitement leading up to a detonation, for example, asking each other “how big the next one is going to be”, with little thought of the potential consequences. 

Participants also described the relatively limited availability of (and access to) knowledge about radiation. Some participants described how there were very few reports in the media about any risks regarding ionizing radiation, nor were any reports accessible to participants during the tests. Of course, this view contrasted across the participants where some veterans perceived this limited access to radiation information as intentional under deception by authorities, which was described in the previous theme. The following extract captures the limited awareness at the time of the tests while referring to the label “guinea pig”:

“Well, how can I put it, I mean don’t forget I said to you earlier, we knew nothing about it. It’s when it all comes to the truth and ii told you I get little booklets and they put stories from the scientists who admitted- And all that carried on. That’s what angers you. When they tell you and you think “you’re a bloody guinea pig”. That’s what gets to you. But there’s nothing you can do about that now. You’ve just got to bite your tongue and that.” (Veteran M).

Collectively, the participants’ young age associated with self-described naivety, attractive location, and the limited access to information regarding the radiation risk explain why most reported no initial concern regarding potential adverse health effects throughout much of their lives. Many of the veterans recalled that their limited concern persisted over approximately the next 20 years after the programme, but subsequent life events appeared to influence the development of the potential psychological impact about perceived ionizing radiation exposure. 

#### 3.3.2. After the Tests

Generally, the veterans stated that they had not encountered health conditions during the first decade or so after the tests, and what appeared more relevant to any psychological impact was their children that they had during the 1960s and 1970s, about which they commented in relation to the media reports emerging later. These veterans remarked that they did not have any worry about their children’s health before they were born, due to limited awareness of health risk. Moreover, in the scenarios where there were health conditions in family members, participants said they initially had no reason to attribute it to any possible exposure because any awareness of other veterans with similar problems had not come to light yet. 

In other participants, the absence of severe health condition and having a healthy descendant prior to the emergence of the reports served as a reassurance that they had not been negatively affected by ionizing radiation exposure. As such, six participants described how they had gone through life with no reported psychological impact relating to the testing programme:

“Well we were very ignorant about it. Didn’t join the nuclear test veterans ‘til about 15 years after did I. so I hadn’t picked up on any things that were appearing in the BNTVA journal till about 15 years after. Then when I got it “well I haven’t got that, haven’t got that, haven’t got that. I’m alright, Jack”.” (Veteran C).

Importantly, any psychological impact experienced was primarily influenced by health-related life events, primarily in family members, and the awareness of reported radiation health effects. This awareness appeared to be facilitated by the formation of the British Nuclear Test Veterans Association (BNTVA) and the emergence of media reports in the 1980s about nuclear testing effects. 

Naturally, most veterans’ awareness of potential adverse consequences began in the 1980s, but for three veterans, this awareness came later than the 1980s since some had not joined the BNTVA until the 2000s, nor had they been exposed to any media reports. Some participants told of their experiences socialising at these meetings where they learned of health conditions in other veterans and their family members, which made them consider their own health. As such, the 1980s appeared to be a central period marked by a change in perceptions as a result of access to radiation-related information at BNTVA meetings (and sometimes by nuclear veteran associations abroad) and media reports. This period appears to be the onset of any psychological impact related to the British nuclear testing programme.

Many participants discussed past experiences and their memories retrospectively and alluded to hindsight. The following extract summarises the theme effectively:

“At the time you think “nah it’s a pretty good posting, we have a great time, have a few drinks in the evening, really easy going. But when you actually get back and things start to occur that hindsight says 20/20 vision. It’s a wonderful thing, and when you get a bit of hindsight things start do come to affect you I think emotionally but I’m quite angry in a way. I’ve got more anger than anything else. Anger and guilt. Anger and guilty, those are the two things. I mean I’m ok mostly I think emotionally but I do get upset sometimes.” (Veteran F).

The above extract highlights that the perceived impact of the testing experience changed over time with reference to different life events and demonstrates that the psychological impact relates to emotions such as anger and guilt. It was clear that the psychological impact was relatively non-existent at the time of the testing programme but appeared to relate to specific events occurring over time, namely the spread of information through the BNTVA and media, interactions with other veterans, and the birth and health development of children and grandchildren. 

## 4. Discussion

Before addressing the qualitative findings, we must first draw our attention to the prevalence of clinically relevant anxiety in this sample. The use of the GAI-SF in this study is the first time a validated measure of anxiety has been used to formalise the extent of anxiety in British nuclear test veterans. Specifically, in this sample, 33.7% met the criteria for clinically relevant anxiety, exceeding the prevalence of anxiety previously thought to exist in this population [35,36,37]. In comparison with older adults elsewhere, 15% of men (older adults, non-veterans) in Forlani et al.’s [44] study screened for probable clinically relevant anxiety (indicated by the GAI-SF). 

Additionally, a mean GAI-SF score of 1.66 was observed in the present exposure worry study. While no GAI-SF data exist for community-dwelling older adults in the UK, elsewhere, a mean of 0.64 in Czech older men aged 60 to 98 [45], 0.68 in Japanese older men aged 60 to 92 [46], and 1.31 in French-Canadian older adults aged 65 to 92 has been observed [47], using their respective translated GAI-SF versions. In this latter study, 25.38% of the sample met the criteria for clinically relevant anxiety [47]. Thus, the present findings indicate an excessive prevalence of clinically relevant anxiety in British nuclear test veterans compared to what could be expected in older men (and older adults generally).

Indeed, anxiety and other mental health issues could be elevated in veterans regardless of involvement in nuclear testing [48], for reasons such as combat stress and relocation [49]. Studies of older veterans elsewhere have reported a generalized anxiety disorder prevalence of 12.0% (mean age = 59.5; [50]), while a pooled prevalence of non-PTSD anxiety disorders was 9.1% according to a recent review [51]. In the upper bounds, a non-PTSD anxiety disorder prevalence of 27.8% has been observed in veterans aged 65 and above [52], but their sample comprised veterans currently receiving mental and nonmental health care. While the cut-off score of 3 or higher for the GAI-SF used in the present study is optimal for detection of generalized anxiety disorder [38], one can speculate that it may detect participants with anxiety-related conditions broader than generalized anxiety disorder, hence the relatively high prevalence of clinically relevant anxiety in this sample. 

**Figure 3:**
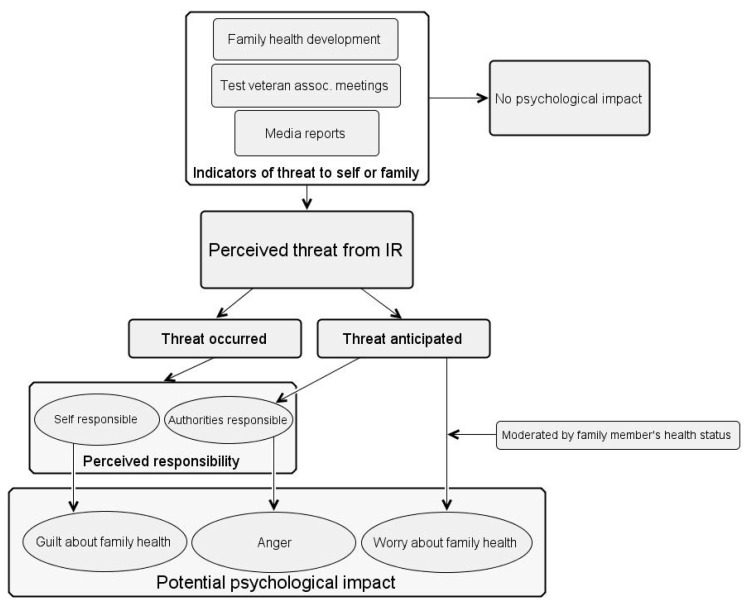
Conceptual model illustrating the potential psychological impact in British nuclear test veterans. The potential psychological impact relevant to the interview participants, namely guilt about family health, anger, and worry about family health. Family health development and health risk information shared through veteran association meetings and media reports serve as indicators of health threats to self or family. Guilt appears to arise out of perceived self-responsibility for threats to family member’s health which have already occurred. Anger may arise out of perceiving authorities as responsible (due to perceived negligence and deception) for past threats and anticipated threats to health. Lastly, worry may arise out of anticipated threats to health, but it is strongly related to family member’s health development.

To explore possible reasons for the anxiety-related mental health issues in this population, the present study included nineteen qualitative interviews (seven of whom met the criteria for clinically relevant anxiety) to explore any worry and the broader psychological impact of the testing programme and how this changed over the life course. In all, the qualitative findings showed three overarching themes to capture the psychological effects associated with involvement in the testing programme, namely “worry, responsibility, and guilt”, “us vs. them”, and “change across the life course”. The qualitative findings are illustrated in a conceptual model below, depicting the potential psychological impact in British nuclear test veterans (Figure 3).

It was generally reported that there was limited impact in relation to worry or anxiety about the health effects of radiation exposure on themselves, but the health effects of descendants was of greater concern, which appeared consistent with the existing literature. For example, while older adults generally report worrying less than younger adults [53,54,55], older adults tend to report worrying more about the health and welfare of loved ones, despite a lower likelihood of worrying about their own health compared with younger adults [54]. For age trends regarding worry content in the context of radiation (following the Fukushima Daiichi disaster), adults aged at least 50 years old were more concerned about the effects of radiation exposure on their future generations, while those of reproductive age (15–49) were more concerned about the delayed effects on themselves [56].

An explanation for why there was limited worry regarding radiation exposure, primarily about their own health, relates to the way worry is conceptualised. Worry is usually conceptualised as negatively valenced thoughts regarding events that might happen in the future [9,57]. Drawing on Tallis and Eysenck’s [12] position that worry is a mental problem-solving mechanism for a possible future outcome, it might therefore be expected that this is more relevant to younger individuals, whereas for those later in life, the future is not regarded as uncertain and worry about their own health might be reduced. Similarly, the veterans’ first-generation descendants had matured and were well into adulthood. Therefore, their children’s future may not be regarded as uncertain, and worry is not as relevant, but their grandchildren’s future remains uncertain, and this worry may then be focused on the grandchild. 

Aside from the participants’ age as a possible explanation for limited worry regarding radiation, another reason may be due to the context of exposure. A considerable amount of literature relates to nuclear power plant accidents where issues such as evacuation and the broader, radical societal change [8,58] may also impact on mental health. However, in the present context, the nuclear weapons tests were anticipated and occurred in relative isolation without issues such as evacuation and social change. While perceived ionizing radiation can be distressing, it is important to unpick different stressors depending on the context to avoid overfocusing the psychological impact to the exposure specifically. 

Separately, as in the Chernobyl accident [8] and US nuclear test veterans [34,59], the present study also sheds light on the role of transparency and accountability from authorities. The perceived negligence, or deception in some cases, and the perceived reluctance for authorities to recognise such negligence or deception may further exacerbate any psychological impact. It was apparent that these sociopsychological issues appeared to manifest as anger and frustration, which highlights the notion of responsibility. Ethical evaluations of risk are strong when the risk is anthropogenic [60,61], and anger is an expected outcome when consequences are to be expected (or have already occurred) and when there is someone else or a group perceived as morally responsible.

Importantly, while the psychological impact was generally limited in those who believed their descendants had not been adversely affected, there was a perceived self-responsibility accompanied with guilt in those who perceived a descendant or wife to have been affected by their exposure. The term “genetic responsibility” has been coined in the medical sociology literature, and could be applied to the present study, but has primarily been explored in the context of genetic screening for cancer-associated genes such as BRCA1/BRCA2. Related to this, the notion of guilt regarding the health of descendants has appeared in qualitative research in men [62,63] and women [64] with BRCA1/BRCA2 mutations. In Hallowell et al.’s [62] study, for example, they similarly describe responsibility to ensure that their descendant does not develop genetic disease. Importantly, Hallowell et al.’s participants generally did not accept blame for being a carrier which contrasts with the test veteran perceptions in this study. While the phrase “just one of those things” was relevant to some of Hallowell et al.’s [62] participants and indeed some of the test veteran participants (particularly prior to the formation of the BNTVA and emergence of media reports), it appears veterans perceive their descendants’ condition as an event caused by the veteran themselves, despite attributing responsibility for the nuclear test event to “the powers that be”.

As such, the present study highlights the need for awareness of relatively unexamined psychological effects of ionizing radiation exposure, specifically guilt, in older adults who may perceive themselves to be responsible for a family member’s illness or, in some cases, death. The importance is that this guilt may add a unique psychological dimension to otherwise normal negative life events (for example, miscarriages and cancers do occur in family members naturally). Indeed, such life events would be psychologically distressing regardless of ionizing radiation exposure, but special attention should be paid to excess psychological consequences resulting from perceived responsibility and subsequent guilt relating to their family members health condition. One can also consider whether any worry prior to the birth of a child or grandchild is in excess in those who perceive their offspring to be at risk of hereditary effects compared to other parents or grandparents. This could apply to other populations exposed to chemical agents, where perceived responsibility for heritable health effects may be relevant.

### 4.1. Limitations

There are possible sample self-selection biases which may skew the prevalence of clinically relevant anxiety in this population. It should be noted that the samples were not randomly selected, but participants interested in a study about worry are more likely to consent to the study following invitation, despite it being made clear that we were also keen to recruit participants who were not worried/anxious. 

While most veterans in the qualitative study reported not being particularly worried, we must be mindful not to understate worry due to potential role of masculinities. In one interview, Veteran E described how women were better at expressing emotion and “opening up” in comparison to men. In addition, input from wives in other interviews provided an indication that there are emotions and possibly a psychological impact which is not being openly expressed by the veteran in the interview scenario. It is known that military culture endorses emotional toughness and stoicism, which appear to overlap with characteristics associated with traditional and hegemonic masculinity traits [65,66,67]. Indeed, Plys et al. [68] postulate that older male veterans may describe anger or frustration instead of sadness as indicators of emotional distress, and one could speculate that this may also apply to worry. Thus, it is possible that being older men and previously working in the military may result in the participants adopting traits such as stoicism, which could affect the emotional expression in the qualitative interviews. 

Separately, the formation of the BNTVA in the 1980s appeared to be a central event where veterans began learning of the potential health effects of ionizing radiation exposure. However, the potential benefit of the BNTVA as a support group was largely unexplored. When life stressors are unique, the support from primary networks (e.g., immediate family and significant others) may be less effective, but the support from secondary networks where they are experientially similar is proposed to buffer the impact of the stressor on mental health [69]. Therefore, the BNTVA (and various veteran organisations) may serve as an effective coping system, especially for individuals who perceived their descendants to have been adversely affected and would be worth examining.

### 4.2. Future Work

While this study focuses on the psychological impact in the veterans themselves, there remains the question of whether their descendants are at risk of any psychological impact of (perceived or actual) paternal ionizing radiation exposure. This question arises following the consideration that adverse health effects could be perceived to be hereditary. Currently, just two studies have examined the potential psychological issues in this regard. Recently, Kamite et al. [70] observed no difference in health anxiety between descendants with atomic bomb survivor relatives and those without atomic bomb survivor relatives, but this measure does not extend to awareness of genetic effects of ionizing radiation exposure. However, an earlier qualitative study observed anxieties in female second-generation atomic bomb survivors focusing on the potential for adverse health effects in their third-generation descendants [71]. Such considerations should be made to descendants of British nuclear test veterans, with a special focus on female descendants since women’s risk of worry is higher than men’s risk of worry in the context of radiological exposure [72].

## 5. Conclusions

This study provides a quantitative indicator of anxiety-related mental health issues in British nuclear test veterans, and elicits a rich account of British nuclear test veterans’ experiences and perceptions with regards to worry and the broader psychological impact. Aside from being a relatively unexamined population, their nuclear testing programme experience is, apart from Japanese atomic bomb events, one of the earliest instances of technological ionizing radiation exposure. Therefore, this study uniquely contributes to our understanding of the psychological impact of perceived exposure over the life course and in later life in men. Any current worry about adverse health effects in themselves was not particularly relevant but worry regarding adverse effects in their grandchildren was certainly applicable to four of the nineteen veterans interviewed. Thus, one can speculate that the anxiety prevalence indicated by the GAI-SF is related to veteran issues beyond any worry specifically about radiation exposure (but may apply to their grandchildren’s health in a few cases). Separate to worry and anxiety, the sense of responsibility for family health and subsequent guilt appeared to be a significant mental health issue, especially in those whose family members had suffered from health conditions. This must be considered in other exposed populations, especially in those who eventually have children since health conditions do occur naturally regardless of exposure to radiological or chemical agents. Moreover, perceived deception and negligence in authorities may conflate any psychological impact associated with perceived exposure and elicit anger, so it is important for authorities to emphasise transparency and accountability when addressing the potential impact on exposed populations.

## Data Availability

The data presented in this study are available on request from the corresponding author. The data are not publicly available due to privacy reasons.
